# Taking things public: a contribution to address human dimensions of environmental change

**DOI:** 10.1098/rstb.2007.0020

**Published:** 2008-02-11

**Authors:** Diógenes S Alves

**Affiliations:** National Institute for Space Research (INPE)CEP 12227-010 Sao Jose dos Campos, Brazil

**Keywords:** Amazon, human dimensions, large-scale biosphere–atmosphere experiment in the Amazon, PPG7, territorial planning, extractive reserves

## Abstract

This paper addresses the question of environmental change in Amazônia, by looking at the experiences of the large-scale biosphere–atmosphere (LBA) experiment in the Amazon, and three other enterprises—the extractive reserves, the Pilot Programme to Conserve the Brazilian Rain Forest (PPG7) and ecological-economic zoning—that address questions of sustainable development in the region. The LBA experience shows how the integration with the social sciences can be critical for science to explore its own outcomes for society, while the other programmes expose environmental change as a problem with too many intersections within society, so the outcomes of any initiative depends on placing it before a complex, tense and wide arena.

## 1. Introduction

Amazônia has received increasing attention in the global environmental change debate due to high annual carbon emissions from deforestation, the interconnections between global climate change and Amazonian ecosystems and losses of biological diversity. As shown throughout this volume, these issues have motivated several research efforts, among which one of the most important is the large-scale biosphere–atmosphere (LBA) experiment in the Amazon, a multidisciplinary effort investigating the functioning of Amazonian climate and biophysical systems, and the effects of changes in land use and climate on such systems (LBA ND*a*,*b*). However, the LBA was designed initially by natural scientists, and the social sciences have always had a marginal position in framing the discussion and directing major lines of inquiry.

This article reviews the creation and evolution of the LBA initiative, and examines why the inattention to social and political considerations in the framework of investigation of environmental change in the Amazon can be risky. Three other examples of initiatives that addressed environmental issues in the region—extractive reserves, ecological-economic zoning (ZEE) and the Pilot Programme to Conserve the Brazilian Rain Forest (PPG7)—further support the need for understanding political and social contexts in proposing either models or solutions to the region's problems. Extractive reserve statutes are an entirely new institution regulating preservation and access to land under a single framework (e.g. Aubertin 1995). ZEE is a territorial planning initiative under which great priority was given to Amazonia (Brasil 1990*a*). The PPG7 programme is a major international cooperation programme funded by the world's wealthiest nations and was directed at preserving biodiversity, reducing greenhouse gas emissions from deforestation and establishing a model of sustainable development (Brasil 1992; World Bank 2000). Each of these initiatives addressed environmental change in Amazônia by adopting different positions for articulation with society, and thus saw different levels of success.

To truly incorporate social factors would mean that environmental change in Amazônia should be discussed in the context of historical social tensions between social classes and sectors, races, genders and regions (e.g. Velho 1976; Schmink & Wood 1984; Aubertin 1995; da Cunha & Almeida 2001). Furthermore, work on the region must acknowledge the recurrent, unresolved demands for ‘economically viable, ecologically appropriate, politically balanced and socially just’ development that have never been met (Benchimol 1989, p. XIV), and the deep-seated forms of social dependence and domination (e.g. Velho 1976; Lena *et al.* 1996) that hugely challenge the discussion of environmental change with society. The empirical elements presented here attempt to show that failure to incorporate these social and political dimensions can lead to the proposal of technical solutions which may be politically unfeasible, and to neglect the intricacies of the process of creating working institutions to face environmental change.

## 2. A short sketch of the LBA experiment and how human dimensions were brought in

The LBA Science Plan proposed to respond to ‘major issues raised by the Climate Convention’, ‘reinforce the Brazilian Integrated National Policy for Legal Amazônia [studying] activities which degrade soil and water resources’, and provide ‘knowledge, in combination with enhancement of the research capacities and networks (…) to devise sustainable alternative land-use strategies along with forest preservation strategies’ (LBA ND*a*). The experiment was established as a multidisciplinary research programme focused on the investigation of the functioning of Amazonian climate and biophysical systems, and the effects of changes in land use and climate on such systems, involving a broad range of disciplines, substantial funding and scientific and technical expertise.

The LBA Science Plan was developed between 1992 and 1995, ‘through a series of … workshops … [and it was] supported … by a number of … institutions’ including the ‘Biospheric Aspects of the Hydrological Cycle, (…) Global Energy and Water cycle Experiment and International Satellite Land Surface Climatology Project’ (LBA ND*a*). The field research began in 1998, and now LBA is in a period of synthesis of results and revision of the Science Plan for the programme's next phase (Batistella *et al.* 2007).

**Figure 1:**
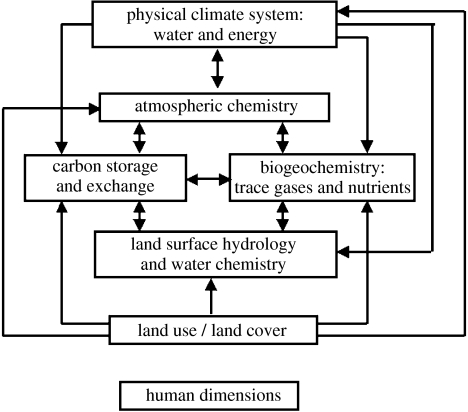
Diagram of the seven components of large-scale biosphere–atmosphere experiment in Amazônia (based on LBA ND*b*).

The Science Plan (LBA ND*a*) is organized around seven components (figure 1). Six of these—physical climate, carbon storage and exchange, biogeochemistry, atmospheric chemistry, land hydrology and water chemistry, and land use and land cover—are the core fields of LBA research; their design was a critical question for the Science Plan development and their articulation—illustrated as arrows in the diagram—nearly correspond to feedback loops in the processes investigated in relation to the two central questions. Studies in individual components were expected to ‘serve their specific disciplinary objectives’ and also contribute to the programme's central questions. ‘To provide a coherent framework for (…) studies that differ widely in subject, location, scale and approach’ components adopt ‘a gradient of land-use intensity and climatic seasonality, (…) [and] a hierarchy of spatial and temporal scales’ (LBA ND*a*). The systems theory paradigm provided a further referential framework for articulation among components and individual studies by offering process parametrization and feedback loops as the basic concepts that helped the dialog among scientists from different disciplines and fields of research.

Most of the LBA model of articulation among disciplines was, in fact, inherited from earlier research of biological, chemical and physical systems whose participants took a leading part in the LBA proposal. Such an arrangement was, in fact, the result of a series of gradual advances in research organization of a very specific kind involving many elements. Looking at the origins of the experiment, Becker *et al*. (2007, p. 7) pondered that its ‘design, questions and epistemology owes a lot to the achievements of other initiatives that preceded it in (…) Amazônia, Africa, North America and Europe. From [them], LBA inherited not only a body of (…) knowledge, but—not less important—networks (…) among scientists from several countries and fields of research’.

The development of the Science Plan included a detailed formulation of research questions for each component; for example, the land-cover/land-use component defined the four following questions:

- What are the rates and mechanisms of forest conversion to agricultural land uses, and what is the relative importance of these land uses?
- At what rate are converted lands abandoned to secondary forests; what is the fate of these converted lands, and what are the overall dynamic patterns of land conversion and abandonment?
- What is the area of forest that is affected by selective logging each year? and
- What are the plausible scenarios of future land-cover change in Amazônia?

These questions illustrate the particular model of articulation among disciplines of LBA: all four questions have direct linkages with the other five ‘systemic’ components in figure 1, related, most particularly, to changes in carbon stocks, water or nutrient cycling. At the same time, these questions also illustrate how the conception of the Science Plan sought to ensure that the component's ‘specific disciplinary objectives’ could be clearly stated. Interestingly enough, these formulations do not explicitly develop the question of coming up with strategies for sustainable land use, but include important elements to account for a diversity of possible land uses, even if no mention is made about the different social and political contexts behind land use. A similar inference could be made from the analysis of the other components: their questions and problems address important issues of climate, and carbon, water and nutrient cycling, although the experiment did not have a systematic approach to explore how its findings could produce an effect on society.

Is it possible that these arrangements could have had peculiar effects on the integration of human dimensions?

### (a) Modelling the Amazon as if people did not matter?

Despite the centrality of the natural sciences in the LBA experiment's conception, the need to incorporate perspectives from the social sciences surfaced repeatedly during the experiment's execution. The LBA Scientific Steering Committee reacted to this by sponsoring several efforts to address the incorporation of human dimensions of environmental change within the programme (e.g. Schor 2005; Becker *et al.* 2007). In addition, the foundation of the International Human Dimensions Programme on Global Environmental Change (IHDP) in 1996 helped to catalyse the idea about human dimensions research within LBA, although ‘tak[ing] social science perspectives on global environmental change’ (http://www.ihdp.uni-bonn.de) presented a challenge for the experiment.

Nobre & Becker (2001) place the incorporation of the seventh, human dimension component into the LBA in December 2000, i.e. well after LBA field research has been already initiated. Referring to it as ‘the human dimensions of Amazônia development’, they use IHDP as a reference for carrying it out, and consider that ‘research on Human Dimensions must necessarily encompass the other six scientific themes’, and ‘the key question (…) in Amazonia [would be] about the dynamics of regional land occupation’. For them, research should include the study of ‘public policies which determine land use in the region’ and ‘attitudes and practices of social agents and sectors in regard to use of natural resources’, and focus on three central issues: ‘(i) deforestation (…), (ii) the restoration of degraded areas (…) (iii) and the consolidation of sustainable productive activities’.

Indeed, most of the human dimensions initiatives during the experiment were linked to the land-cover/land-use component, whose original formulation had already focused on deforestation and land/forest degradation. The four questions of this component were aimed at the articulation with the other five systemic components, in particular, at changes in carbon stocks, water and nutrient cycling. Yet, they also include concepts whose definition would be difficult within the strict limits of the natural sciences: concepts like mechanisms of forest conversion (question i), land abandonment or patterns of land conversion and abandonment (question ii) depend on the social sciences to be fully developed; the same would apply to investigating the relative importance of different land uses (question i), selective logging (question iii), or the plausibility of different scenarios of land-cover change (question iv).

From this, it may be suggested that LBA proponents were able to incorporate important concepts of land use from the social sciences into the Science Plan. At the same time, however, such concepts were not formulated during conceptual and methodological discussions comparable to other topics of the Plan, generally oriented towards the problems of water, carbon and nutrient cycling. It is suggested that this occurred owing to the absence of social scientists among the proponents of the experiment: in fact, LBA (ND*b*) lists a large majority of the Science Plan contributors in the natural sciences, system theory/mathematics or remote sensing techniques.

Meanwhile, LBA execution—based on open, competitive calls for proposals for its greater part—contributed to a gradual growth in articulating with the social sciences, to the measure that it stimulated collaborations with social scientists both responding to the original LBA questions and suggesting new perspectives of investigation. Also, the periodic and frequent meetings of different types—like the Steering Committee meetings, LBA scientific conferences and LBA/ECO meetings—which were instrumental to the exchange of ideas among LBA participants (Schor 2005), allowing, in particular, a reassessment of LBA questions in the face of the very dynamic nature of the land-use change processes (e.g. Asner *et al.* 2006; Morton *et al.* 2006; Alves 2007*a*), even if these advances were confined to LBA original objectives for the most part.

Another aspect of the problem is related to the perception of the very concept of human dimensions among LBA participants. One prevailing position was that a human dimensions component would be a place for social science studies, according to the model that research components were to ‘serve their specific disciplinary objectives’. In fact, the Scientific Steering Committee recognized the relevance of the social sciences, sponsoring a survey of social science production on Amazônia (Becker 2007), and a workshop to debate gaps of knowledge in that field (Becker *et al.* 2007), emulating the 1992–1995 strategy to implement the human dimensions component. In addition, the Committee took the initiative to bring in a few social scientists.

Still, Schor (2005) observed different perceptions on the subject among LBA participants, including a view that human dimensions were already included in LBA ecological studies, another emphasizing economic research, another proposing to investigate territorial patterns, and opposing paradigms related to land degradation and land-use intensification (see also Mortimore 1993; Faminow 1998). In addition, the views of the institutional field about human dimensions research have been contradictory: a review panel appointed by the Brazilian Ministry of Science and Technology made a negative evaluation of the small number of human dimensions projects in LBA, stating that this indicated a ‘modest treatment of the demographic dynamics and social and environmental impacts’ (Philippi Junior *et al*. 2003), but Schor (2005, p. 122) found that, for that same Ministry, ‘deforestation should be studied by Brazilians, not by international programmes like LBA’.

Another interesting issue is the lack of systemic connections, depicted as arrows, between the human dimensions and the other six LBA components in figure 1. The origins of this representation has various interpretations among LBA participants, including the recognition of the different (non-systemic) nature of the human dimensions, or the actual depiction of human dimensions being the ultimate question permeating environmental change research. Again, the lack of social scientists in LBA conception, in association with the LBA model based on the systems theory paradigm, limited the latitude of such discussions, despite an open approach to broaden the experiment scope and agenda.

Systems theory aims at the representation, simulation and/or control of a broad variety of processes ranging from engineering to biology, ecology and social systems. Its approach is based on decomposing a process into component sub-processes, and specifying how different sub-processes interact with one another, providing a powerful tool for the articulation of disparate disciplines in a wide range of problems, as exemplified in the case of LBA's six ‘core’ components (figure 1). Notwithstanding the vast possibilities of systems theory, a range of commentators has questioned, most particularly, its possibilities to approach the tensions and contradictions within the society (see Heidegger 2000 (1954); Habermas 2000 (1968); more recently, Whiteside 1998; Leff 2002; Mirowski 2003). In effect, although systems theory can be useful to social sciences, it offers a limited background to formulate questions related to social stratification, dependence, domination, agency, values, attitudes, anomy and other categories that permeate the social and political background against which Amazônia and environmental change are discussed.

The LBA constituency also seems to have influenced the perception of its potential outcomes for society by its proponents and participants. If the proposal correctly identified its main contributions to society in the fields of the climate convention and sustainable land use, then the background behind these formulations was not subjected to the discussions dedicated to the other—‘natural science’—questions.

The inattention to the need of conceptual discussions about the statement of LBA outcomes for society reflects the different perspectives of the natural and social sciences. In fact, despite the clear relevance of LBA research for key aspects of the Climate Convention (e.g. articles 5, 6 and, most importantly, 2) and for providing subsidies for sustainable land use (particularly in respect to its agro-ecological dimensions), the proposal does not elaborate, for example, on the political aspects of the Climate Convention and its negotiations (e.g. Viola & Leis 2001; Lahsen 2005), or on the discussion of the very notion of development or sustainability (e.g. Martins 1976; Furtado 1996; Montibeller Filho 2004). More generally, in the field of the natural sciences, the statement of outcomes of research for society may not be exposed to the kind of questionings formulated in the social sciences, like the recurrent enquiries about the nature or the role of science (e.g. Giddens 1996; Latour 2000; Martins 2002; Moraes 2002), the very nuanced epistemological debate (e.g. Latour 1999; Leff 2002; Moraes 2002), or the questions about the very neutrality of science to address societal issues (Schor 2005). Finally, the narrowness of LBA perspectives may also be related to its participants generally taking more of a ‘bio-environmentalist’ stance to environmental change, a frequent although not a hegemonic position within the scientific field (Hogan & Tomasquim 2001; Alonso & Costa 2002). In this case, the contribution of the social sciences could help in discerning different stances and articulations taken within this field, and their implications for an ampler understanding of the outcomes of research for society.

It can be proposed that higher levels of integration between the natural and social sciences will require the assimilation of all these doubts, debates and their various shades. The risk of excluding social and political considerations in addressing environmental change—and, in the end, in attempting to influence environmental policy—can be further explored by looking at three other projects, as proposed below.

## 3. Running aground on social reality: multiple stakeholders and the need to discern them

This section draws on elements from existing analyses about the PPG7, ZEE and extractive reserves to illustrate how complex the social and political background related to environmental change can be in Amazônia. The PPG7 programme is an international cooperation programme directed at preserving biodiversity, reducing greenhouse gas emissions from deforestation and establishing a model of sustainable development funded by the G7 countries (Brasil 1992; World Bank 2000; Mello 2006). ZEE offers a broad foundation for territorial planning at the national, regional, state and municipal levels, by prescribing land use based on technical assessments and political negotiations (Brasil 1990*a*; Mahar 2000; Lima 2006; Mello 2006). Extractive reserves are a modality of concession ‘dedicated to the self-sustainable extraction and conservation of renewable natural resources by extractivist populations’ (Brasil 1990*b*).

In contrast to the LBA experiment, these experiences included the articulation with a multiplicity of stakeholders motivating the examination of their performance from the perspective of the varying social and political conditions in Amazônia; also, they included key stakeholders outside Amazônia and even outside Brazil. Using part of several studies covering a considerable range of themes, the analysis is limited to a few lessons illustrating the implications of facing a multiplicity of social and political contexts when environmental issues are at stake.

ZEE can serve well to delineate the complexity of facing multiple interests and motivations behind environmental questions in Amazônia. Ecological zoning has often been proposed as a technical solution for territorial planning in Brazil—frequently based on principles of pedology, geomorphology and agronomy. However, analysing zoning in the state of Rondônia, Mahar (2000, p. 126) found that ‘ [f] armers, ranchers and loggers have all, to some extent, reacted negatively to [zoning] restrictions, because many of the environmental and social benefits resulting from leaving the forest intact (…) accrue to stakeholders located outside the state and even the country’. This view does not imply that these stakeholders opposed *any* kind of zoning but, rather, neglecting to address critical political aspects imperilled the proposal significantly. In a cross-analysis of the PPG7 programme and zoning, Mello (2006) considered that while the Brazilian Federal Government emphasized a technical solution for zoning focusing on agricultural expansion, prescribing conservation to areas of higher risk of erosion, foreign PPG7 funders had hugely different and often conflicting positions directly connected to financing zoning: Germany required its funds to support conservation and not economic development, while the UK stipulated that zoning be participatory and reflected aspirations for development of each community; the World Bank was inclined to promote the model implemented in Rondônia and Mato Grosso, but, at times, supported the German position too. Its study purports that state-level implementation actually depended on the origins of funding, resulting on the lack of an effective integrated strategy for environmental management.

From another perspective, extractive reserves provide an example of local stakeholders supporting preservationist goals, although with a range of motivations behind this support. Aubertin (1995) places the creation of extractive reserves in the context of increasing rural violence associated with frontier expansion and the decline of the traditional rubber economy framed by the *aviamento* form of domination. Da Cunha & Almeida (2001) argue that, during the conception of the extractive reserve model, the background problem remained, for rubber tappers, about agrarian and labour problems, implying that rubber-tappers’ support for these statutes were not dissociated from the fragility of institutions assuring access to land and effective coercion of rural violence.

The variety of articulations and interests involved in pro-environment advocacy also appear in several other analyses. Little (2004) argues that different social groups in Amazônia can assimilate the environmentalist agenda, adapting it to their cosmology and objectives. He distinguishes six different environmentalist orientations with differing relations to the social movements; PPG7 is an example of the ‘techno-environmentalist’ orientation that emphasizes project-oriented, technical solutions to environmental problems, an approach strange and even harmful for certain social groups; another orientation, ‘socio-environmentalism’, took an active part in the conception of the extractive reserve statutes and generally acts in concert with social movements targeting strategic political issues. Another author, Lima (2006), sees diffuse, not necessarily convergent, and at times opportunistic, interests in the ‘construction of social environmentalism’.

Preservationist projects and environmental problems have often stimulated a high degree of mobilization in the fields of science and technology. Yet, analyses of the performance of these fields alert to the challenges of effective articulation with society to face such problems: for example, the contributions of the PPG7 science and technology subprogramme to PPG7’s overall goals are perceived as modest, even if the quality of research has not been questioned (World Bank 2000); Mahar's (2000) analysis of ZEE in Rondônia considers the most critical problems to be political in nature, suggesting that technical aspects were, to some degree, overemphasized. Such mobilization can assume different forms: for example, PPG7 tended to take more of a preservationist perspective, while zoning tended to privilege upon technical aspects more relevant for agriculture. It can also have, at times, critical social and political implications: Mello (2006), for example, shows that endless discussion of zoning technical issues promoted by the Federal technocracy delayed critical political negotiations, while other territorial policies favouring economic development were put forward.

Finally, the most important outcome of the three analysed initiatives can be found in their contribution to the establishment of a public sphere to debate the environmental question in Amazônia, as some stakeholders have stepped into a public sphere for the first time, although this process exposed several conflicts and contradictions. Furthermore, this process has also been a vital aspect of the institutionalization of the environmental question in the region and in Brazil. This understanding can be one key element to be taken in consideration when pondering future developments and the fate of Amazônia.

## 4. Conclusion

Science and technology provide critical elements to understand environmental change, but, as the LBA experience shows, the lack of close articulation with the social sciences misses important issues, diminishing the potential impacts of that understanding. In due course, that understanding has to be put into use by society itself, and the social sciences are key to understand this complex process, to help in making science results public, and exploring how society articulates publicly, most particularly, in search of institutional responses to change. In that context, existing analyses about the institutionalization of the environmental question in Brazil can provide further insights on the conflicts and articulations that accompanied this institutionalization. To conclude, I shortly review some of these findings, to offer few topics that may be relevant to expand these discussions in the future.

For Alonso & Costa (2002), the institutionalization of the environmental question in Brazil has been accompanied by the submission of the environmental debate to administrative rationality. Carvalho & Brussi (2004) show how this contributed to enlarge the distance between the social and environmentalist movements and increase the tension between them; they note the different articulations of the social and the environmentalist movements, the former having a significantly weaker position in its capacity of articulation within the Brazilian state and internationally. These points seem important to understand further the social and political contexts in which Amazônia is debated, but have not been broadly considered by academia (Alonso & Costa 2002; Alves 2007*b*).

From another perspective, that institutionalization may include not only the conflicts and contradictions described so far, but also some degree of *accommodation*, in the sense that the antagonism involving a variety of interests may have gradually diminished. Indeed, the passing of a broad range of legislation and the creation of environmental agencies reveal an important change in the political field in comparison with the 1970s and the 1980s, when environmental problems mostly mobilized opponents to the military regime (e.g. Carvalho & Brussi 2004). On the other hand, such advances can be contrasted with the discretion in debating alternative development models, the relative weakness of environmental institutions and the paradoxical trajectory of environmentalist mobilization in Brazil, which gradually shifted from local to global environmental issues (e.g. Simões 2001; Tesh & Paes Machado 2004).

It is suggested that this state of affairs could be associated with the delineation of a new estate (*estamento*), where such sociopolitical groups represent a key category recognized in Brazilian political practice (Martins 1976; Faoro 1987). In this respect, a few other postulations (see Ianni (1973) for theoretical frameworks) can be of help to assess the evolution of the environmental question. First, helped by a ‘conscience of status’ shown by large numbers of environmentalists, this estate is capable of mobilizing relatively broad support in society. Second, while the legitimacy of such an estate would be based on its capacity to advance the environmental agenda, its ability to survive harsh political infighting would be aided by strong and visible global alliances, justifying, to a certain degree, a gradual shift in the Brazilian environmentalist movement, while helping the estate's recognition as a contender in the political field. Third, the structure of this estate would not be monolithic; in particular, techno-environmentalists stances might be privileged from their articulations with the technocracy and fields of science and technology, and from the ‘naturally’ key role assumed by technical issues in environmental hearings and proceedings (such articulations, however, are not to be confounded with unconditional alliance, owing to the differences within and among all of these estates).

The final point is that discussing future frameworks to address issues of climate change and sustainable development in Amazônia can greatly depend on learning from the initiatives and the settings discussed here. In addition, the very agenda of research addressing these issues may increasingly depend on the effective recognition of the different positions involving the fields of science and technology and the different actors to face the societal problems of environmental change.
